# Anxiety symptoms, rule learning, and cognitive flexibility in non-clinical psychosis

**DOI:** 10.1038/s41598-022-09620-z

**Published:** 2022-04-05

**Authors:** Jadyn S. Park, Katherine S. F. Damme, Franchesca S. Kuhney, Vijay A. Mittal

**Affiliations:** 1 Department of Psychology, Northwestern University, Evanston, IL USA; 2 Department of Psychiatry, Northwestern University, Chicago, IL USA; 3 Institute for Innovations in Developmental Sciences (DevSci), Northwestern University, Evanston, Chicago, IL USA; 4 Department of Psychology, University of Illinois at Chicago, Chicago, IL USA; 5 Medical Social Sciences, Northwestern University, Chicago, IL USA; 6 Institute for Policy Research (IPR), Northwestern University, Chicago, IL USA

**Keywords:** Psychology, Human behaviour

## Abstract

Individuals with psychotic-like experiences (PLEs) represent a critical group for improving the understanding of vulnerability factors across the psychosis continuum. A growing body of literature has identified functional deficits associated with PLEs. However, it is unclear if such deficits purely reveal the underlying psychosis vulnerability or if they are also linked with comorbid anxiety symptoms. Although anxiety disorders are often associated with impairments in psychosis-risk, symptoms of anxiety may facilitate executive functioning in certain psychosis groups. The Community Assessment of Psychic Experiences was completed to assess psychosis-like symptoms in a total of 57 individuals, and its median score was used to categorize PLE groups (high-PLE = 24, low-PLE = 33). Anxiety symptoms were measured via the Beck Anxiety Inventory, and cognitive flexibility was measured by the Penn Conditional Exclusion Test. The high-PLE group endorsed more anxiety symptoms, demonstrated poorer accuracy and efficiency on the cognitive task, and made more perseverative errors compared to the low-PLE group. Within the high-PLE group, higher levels of anxiety symptoms were associated with better performance and less perseverative errors compared to individuals with lower levels of anxiety symptoms. Conversely, greater anxiety symptoms were associated with poorer performance in the low-PLE group. Taken together, these findings provide a preliminary support for a potential psychosis vulnerability × anxiety symptom interaction. Given the interest in the psychosis continuum and potential treatment implications, the present findings warrant replication efforts.

## Introduction

Cognitive deficits are often observed in individuals with psychotic disorders along a spectrum^1^. These observed cognitive deficits impact executive functioning, as displayed by mental rigidity, difficulty with rule learning, and perseverative errors^2–4^. However, it is not known if such impairments extend to non-clinical psychosis (NCP) population; that is, non-help seeking individuals with brief, psychotic-like experiences who are otherwise healthy^5^. It also remains unknown whether such executive functioning deficits are related to the levels of psychotic-like experiences (PLEs) or to co-occurring symptoms, such as anxiety. Anxiety diagnoses are highly comorbid in psychosis disorders^6^ and are related to deficits in cognitive flexibility and rule learning^7^. Co-occurring symptoms contribute to cognitive decline in individuals with higher PLEs. However, anxiety symptoms may be beneficial to executive functioning in early stages of psychosis^8^ or reflect an intact executive system. As a result, it remains unclear how anxiety symptoms may impact executive function in individuals with PLEs.

Executive function is closely involved in our day-to-day activities and is predictive of a range of functional outcomes, from social-emotional competence to occupational functioning^9,10^. Deficits in executive functioning have been widely observed in individuals across the psychosis continuum, including: (1) individuals with schizoaffective disorders^11^, (2) those who are at clinical high-risk for developing psychosis^12^, and (3) those who have experienced first-episode psychosis^13,14^. Even in the absence of psychotic symptoms, positive psychosis-like experiences are prevalent among the general population^15^, and experiences such as perceptual aberration and superstitious beliefs have been associated with impairment in executive functioning without general cognitive deficits^16^. Indeed, those with PLEs provide a useful construct that represents common subclinical experiences that are associated with a broad continuum of functioning. It remains unknown, however, whether such executive deficits in NCP individuals are derived as a function of PLEs independent of the confounding phenotypes associated with psychosis.

Community-based surveys reveal that PLEs are associated with higher prevalence of anxiety disorders even in the general population. One out of four young adults with hallucination or delusion-like experiences report having comorbid anxiety disorders^17^ and nearly 40% of individuals on the psychosis spectrum have co-existing anxiety disorders^18^. Despite such high rates of comorbidity, little work has examined the independent or shared correlates of anxiety symptoms and PLEs on executive functioning. Critically, co-existing anxiety symptoms in NCP individuals may not only affect executive functioning but also multiple domains of life, such as global functioning, emotion processing, and mental health outcomes^19,20^. Thus, it is important to elucidate the role of anxiety symptoms in emerging psychosis symptoms.

Anxiety symptoms in the context of PLEs may have an additive detrimental effect on cognitive functioning. Core features of anxiety disorders such as rumination and mental rigidity^21,22^ can negatively impact executive function (e.g., decreased cognitive flexibility and rule learning). Indeed, studies have found that anxiety symptoms were predictive of perseverative errors and less accurate and efficient responses in neurocognitive tasks in anxious individuals^23,24^. However, emerging evidence suggests that anxiety disorders in psychosis may be largely unrelated to cognitive performance^25^ and that symptoms of anxiety may even provide benefits to some aspects of cognitive functioning (e.g., verbal fluency)^26^. Especially in non-clinical samples, greater levels of positive symptoms have been correlated with better performance in working memory and verbal/visual learning^27^. Thus, it has become important to consider how anxiety symptoms interact with PLEs and whether such interaction has a positive effect on other aspects of executive functioning such as cognitive flexibility and rule learning.

The beneficial effects of anxiety symptoms on executive functioning have been described in two existing hypotheses: the optimal arousal theory and the adaptation theory. Evolutionary psychology suggests that non-clinical levels of anxiety have proven valuable for human safety and functioning^28^. Yerkes and Dodson^29^ have extended this into the optimal arousal theory, stating that a certain level of arousal is necessary for the most effective learning. They suggested an inverted-u shaped curve to describe learning as a function of arousal. This model implies that either insufficient or excessive levels of anxiety symptoms may result in learning deficits. Psychosis symptoms are often observed with lower levels of autonomic arousal during baseline relative to healthy individuals^30,31^, and greater parasympathetic arousal, a physiological response associated with heightened anxiety symptoms, relates to cognitive performance among individuals on the lower end of the psychosis spectrum^32^. Thus, based on the optimal arousal theory, it may be supposed that deficient anxiety symptoms may be one of the ingredients that contribute to the cognitive impairment among psychosis-risk population. In other words, some level of anxiety symptoms may provide an optimal level of arousal for better performance in NCP population.

In contrast, adaptation theory supposes that greater levels of anxiety symptoms are a *marker* of intact cognitive functioning, rather than a cause for impairment^8,33^. Some researchers hypothesize that individuals who have experienced a stressful event (such as a recent psychosis-like experience) but with intact cognition have greater insight of their stressful mental state, leading to increased levels of anxiety symptoms—an adaptive response given the event. Indeed, studies have found that greater anxiety symptoms were associated with better cognitive performance in first-episode psychosis, providing further support for the theorized model. These findings make an interesting case as patients on the psychosis spectrum with comorbid affective or substance use disorders, rather than a comorbid anxiety diagnosis, report negative cognitive outcomes^34^. The proposed beneficial effects of anxiety symptoms on executive functioning in psychosis warrant further exploration of this phenomenon in the community members experiencing psychosis-like symptoms.

The present study compares executive functioning performance in individuals with high and low levels of PLEs and explores the potential correlates of anxiety symptoms. Components of executive functioning, cognitive flexibility and rule learning, were examined in terms of accuracy, efficiency, and perseverative errors, using a computerized task that is widely used across healthy and clinical populations^35,36^. The current study examined if individuals with high levels of PLE (high-PLE group) would demonstrate poorer task performance than those with low levels of PLE (low-PLE group). We hypothesized that the high-PLE group were likely to make less accurate and efficient responses and more perseverative errors in a cognitive task, relative to those in the low-PLE group, based on similar findings in individuals at clinical high-risk for psychosis^12,37^. In exploratory analyses, the current study then tested whether such group differences in task performance were correlated with anxiety symptoms.

## Methods

### Participants

Sixty-six young adults (ages 17–24; *M* = 20.38, *SD* = 2.00) in the Chicago area were recruited through the Adolescent Development and Preventative Treatment (ADAPT) program. Participants were recruited from a range of sources, including those from the general community, as well as participants from a subject pool at a private midwestern university. The participants were administered the Community Assessment of Psychic Experiences (CAPE; Stefanis et al.^38^) and the Beck Anxiety Inventory (BAI; Beck et al.^39^) to assess their psychosis and anxiety symptoms. CAPE score from the Positive Frequency dimension was used to classify them as either high-PLE (score 9 or above; *n* = 29) or low-PLE (score 8 or below; *n* = 37), where the high-PLE group was characterized as those who endorsed high levels of psychotic-like experiences (Table 1). The cutoff score for this study was adopted from a larger sample (*n* = 246) screened from the noted subject pool recruitment source, where 9 was the median score. Participants also completed the Penn Conditional Exclusion Test (PCET), a well-validated neurocognitive test that measures executive functioning. Nine out of 66 participants were excluded from the analysis due to missing data (high-PLE = 24, low-PLE = 33). The CAPE and the PCET were completed on the same day, during the experimental visit. The BAI was completed within a month of their experimental visit.

**Table 1:** Demographic characteristics.

	High NCP *n* = 29	Low NCP *n* = 37	Group differences
**Gender**			NS
Male (%)	6 (20.7%)	10 (27.0%)	
Female (%)	22 (75.9%)	26 (70.3%)	
Unknown (%)	1 (3.4%)	1 (2.7%)	
**Age**			NS
Mean years (SD)	20.79 (1.95)	20.06 (1.97)	
**Education**			NS
Mean years (SD)	14.46 (3.20)	14.52 (1.84)	
**CAPE**			*p* < .05
Mean score (SD)^a^	13.72 (3.57)	4.22 (2.51)	
**BAI**			*p* < .05
Mean score (SD)	15.96 (9.77)	11.31 (8.20)	

^a^CAPE frequency score from positive domain only.

### Ethics approval statement

The current study was approved by the Institutional Review Board (IRB) at Northwestern University. All participants provided informed consent. All experiments were performed in accordance with institution-approved guidelines and regulations.

### Clinical assessments

The Community Assessment of Psychic Experiences (CAPE) is a 42-item self-report questionnaire that measures psychotic-like experiences in the general population^38^. The questionnaire assesses positive, negative, and depressive symptoms of psychosis and is consisted of 20, 14, and 8 items, respectively. For each item, participants indicate the frequency of the symptom on a 4-point Likert scale (“never”, “sometimes”, “often”, “nearly always) and the level of distress (“not distressed”, “a bit distressed”, “quite distressed”, “very distressed”). For this study, frequency scores from only the positive symptom domain were taken into account to identify the NCP groups, as the 20-items have been validated to identify individuals at clinical high-risk for psychosis^40–42^.

The Beck Anxiety Inventory (BAI) is a 21-item self-report questionnaire used for measuring anxiety symptoms^39^. The questionnaire assesses subjective (e.g., terrified or afraid, nervous) as well as somatic (e.g., numbness or tingling, hands trembling) sensations associated with anxiety. Participants are asked to rate how much they were bothered by each item within the past month, including the day of the assessment. For each item, participants indicate the severity of the symptom on a Likert scale from 0 to 3. With high internal consistency (Cronbach’s alpha = 0.94), numerous studies have demonstrated its utility in assessing anxiety symptoms in both clinical and non-clinical population^43–45^.

### Task description

The Penn Conditional Exclusion Test (PCET) is a computerized task that measures cognitive flexibility and rule learning by utilizing an “odd-man-out” paradigm^36,46,47^. The PCET measures set shifting and perseverative errors, akin to the Wisconsin Card Sorting Test^36^ and has been widely used to assess executive functioning in individuals with psychosis^35,48^. The metrics provided by the PCET are well-validated to capture dimensions of executive control^47^, and numerous studies have found significant performance differences in various clinical populations from that of healthy controls^36,49,50^. In this task, participants are presented with four objects on the screen that are different from each other by one of three categories: size, shape, and line thickness. The participants are asked to select the object that does not belong with others (i.e., does not fit the category) and are given feedback (“correct”/“incorrect”) upon response. After 10 consecutive correct responses, the category changes and participants are expected to learn the new rule based on the feedback. The main outcome variables of interest in this study were accuracy, efficiency, and perseverative errors. Accuracy was defined as the proportion of correct trials out of total trials: (category achieved + 1) * (correct responses/total responses). Efficiency reflected the average of accuracy and speed: accuracy/log(RT of correct responses). Perseverative errors were defined as the number of incorrect trials in which the error was due to a repetition of a previously made error despite corrective feedback. All variables were automatically generated by the Penn Computerized Neuropsychological Test Battery. Task performance was measured on the basis of accuracy and efficiency^36,47^. Detailed explanations of how the variables are derived are described in Gur et al.^51^ and Moore et al.^47^.

### Analytical strategy

The present study examined whether the performance in the PCET (accuracy, efficiency) and perseverative errors varied by PLE group, and whether anxiety symptoms related to group differences in performance and perseveration errors. All analyses and visualizations were completed using RStudio version 1.4.1106. First, a student’s *t* test was used to test the group differences in symptoms of anxiety and psychosis between high-PLEs and low-PLEs. Then, another student’s *t* test was used to examine group differences in PCET accuracy, efficiency, and perseverative errors. Next, three separate general linear models examined how each executive function metric (PCET accuracy, efficiency, perseverative errors) was correlated with anxiety symptoms (total BAI score) and PLE group status (high-/low-PLE). Post-hoc group comparisons were restricted to significant findings in accordance with protected Fisher’s Least Significant Difference test, using the logic provided by Fisher’s protected tests to preserve sample power^52^.

## Results

### Participants

**Figure 1:**
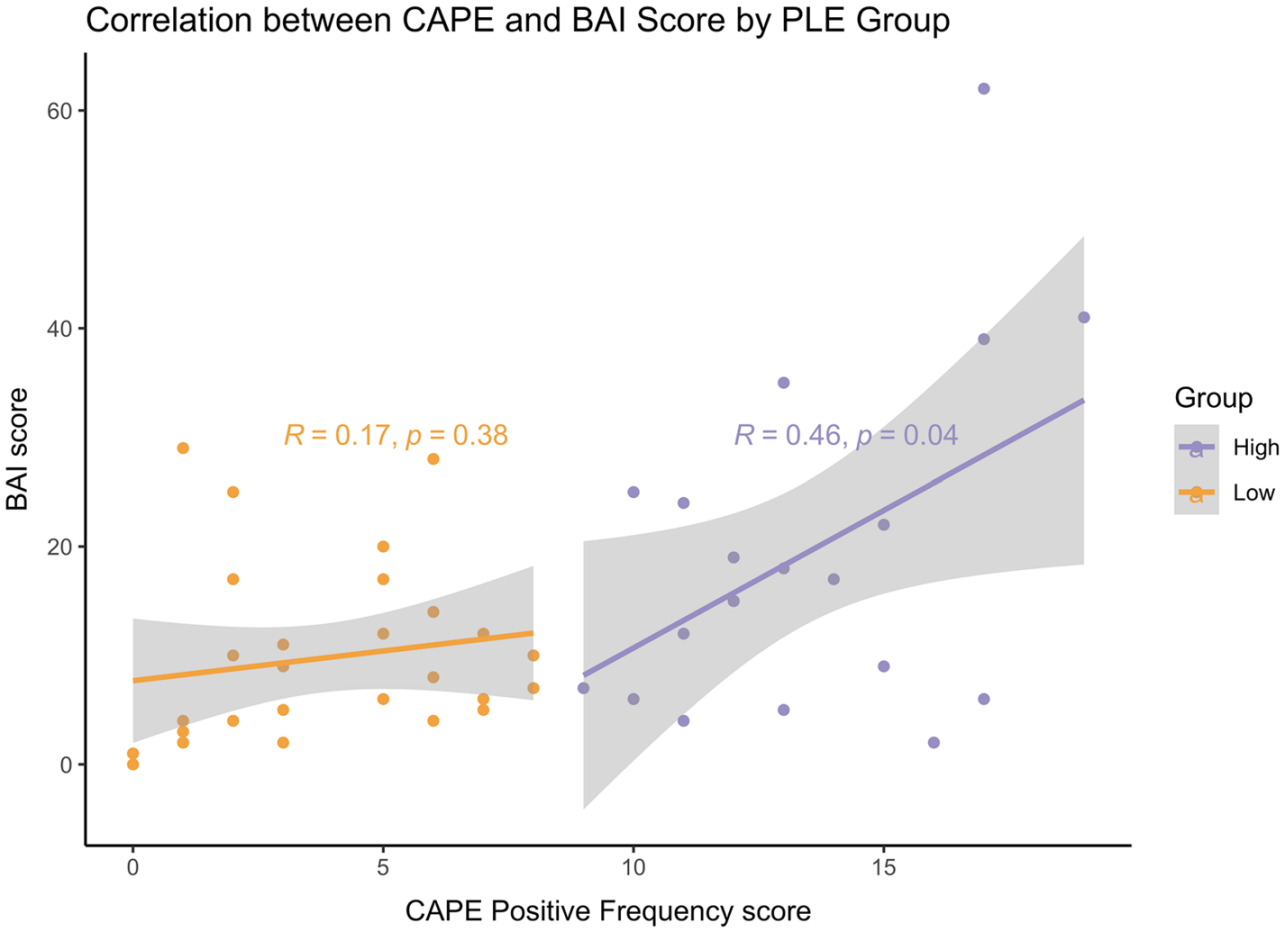
Correlation between PLEs and anxiety symptoms by PLE group. There was a significant positive correlation between PLEs and anxiety symptoms within the high-PLE group only. *BAI* Beck Anxiety Inventory, *CAPE* Community Assessment of Psychic Experiences, *PLE* Psychotic-Like Experiences.

There was no significant difference in sex (${\chi }^{2}$(2,57) = 0.37, *p* = *0.8*32), age (*t*(55) = 1.09, *p* = 0.28), or education level (*t*(29.37) = 0.23, *p* = 0.82) between the two groups. As expected, by virtue of the grouping strategy, the high-PLE group showed significant PLE elevations (*M* = 13.72, *SD* = 3.57) compared to the low-PLE group (*M* = 4.22, *SD* = 2.51); *t*(48.18) = 12.17, *p* < 0.01. There was a significant main effect of group status on anxiety symptoms, *F*(1,53) = 6.86, *p* = 0.01, such that the high-PLE group scored significantly higher on the BAI (*M* = 15.96, *SD* = 9.77) than the low-PLE group (*M* = 11.31, *SD* = 8.20) (Table 1). In general, there was a positive correlation between PLE and anxiety symptoms (pearson’s *r* = 0.48, *p* < 0.01), which was driven by individuals in the high-PLE group (pearson’s *r* = 0.46, *p* = 0.04) not the low-PLE group (pearson’s *r* = 0.17, *p* = 0.38), Fig. 1. Group differences in the executive task performance is summarized in Table 2. The anxiety symptoms are illustrated as a continuous variable in the supplementary materials.

**Table 2:** Performance summary.

	Accuracy	Efficiency	Perseveration
High-NCP	2.18 (.92)	.21 (.1)	15.33 (9.63)
Low-NCP	2.75 (.76)	.28 (.08)	9.09 (6.49)
*p*	.014*	.012*	.009**
Cohen’s D	.684	.694	.785

Mean and standard deviation value of accuracy, efficiency, and perseveration errors by group. In all facets of executive functioning measures, the high-NCP group showed deficits compared to the low-NCP group. *p* < .05*; *p* < .01**.

### Group differences in executive functioning

#### Accuracy

**Figure 2:**
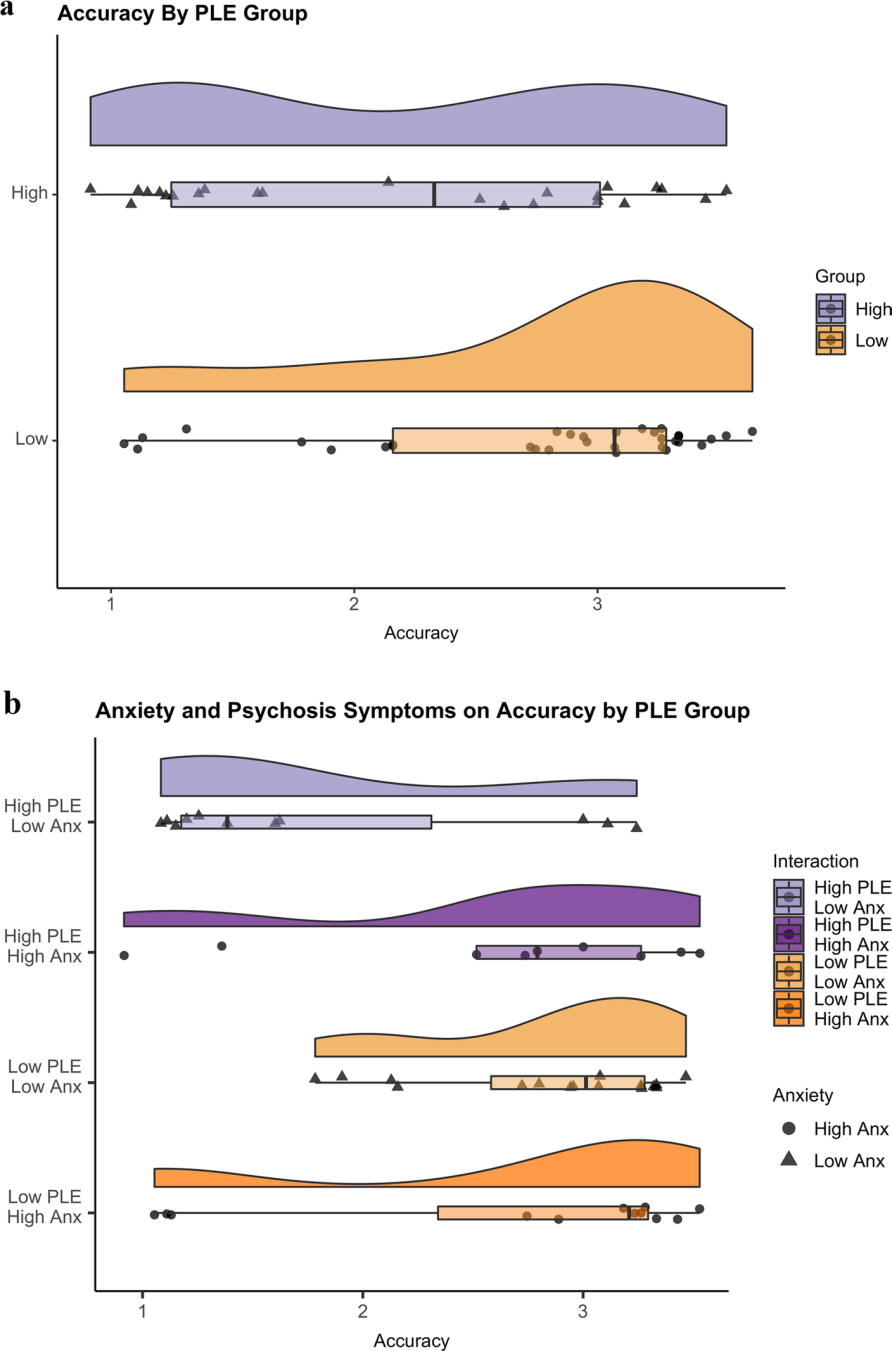
(**a**) Accuracy by PLE group*.* Each dot represents the value from each individual. The distribution of values is indicated by the density plot as well as the box plot. Overall, the high-PLE group made significantly less accurate responses compared to the low-PLE group. *PLE* Psychotic-Like Experiences. (**b**) Accuracy relates to anxiety symptoms in the high-PLE group. Individuals with greater levels of anxiety in the high-PLE group made accurate responses comparable to those with low-PLE. Note that the PLE groups were divided into different high/low anxiety groups for illustrative purposes only and all analyses treated anxiety symptoms continuously. *Anx* anxiety symptoms, *PLE* psychotic-like experiences.

Accuracy was significantly different between the two groups, such that the high-PLE group was less accurate (*M* = 2.18, *SD* = 0.92) than the low-PLE group (*M* = 2.75, *SD* = 0.76): *t*(55) = 2.55, *p* = 0.01, *d* = 0.68 (Fig. 2a).

#### Efficiency

**Figure 3:**
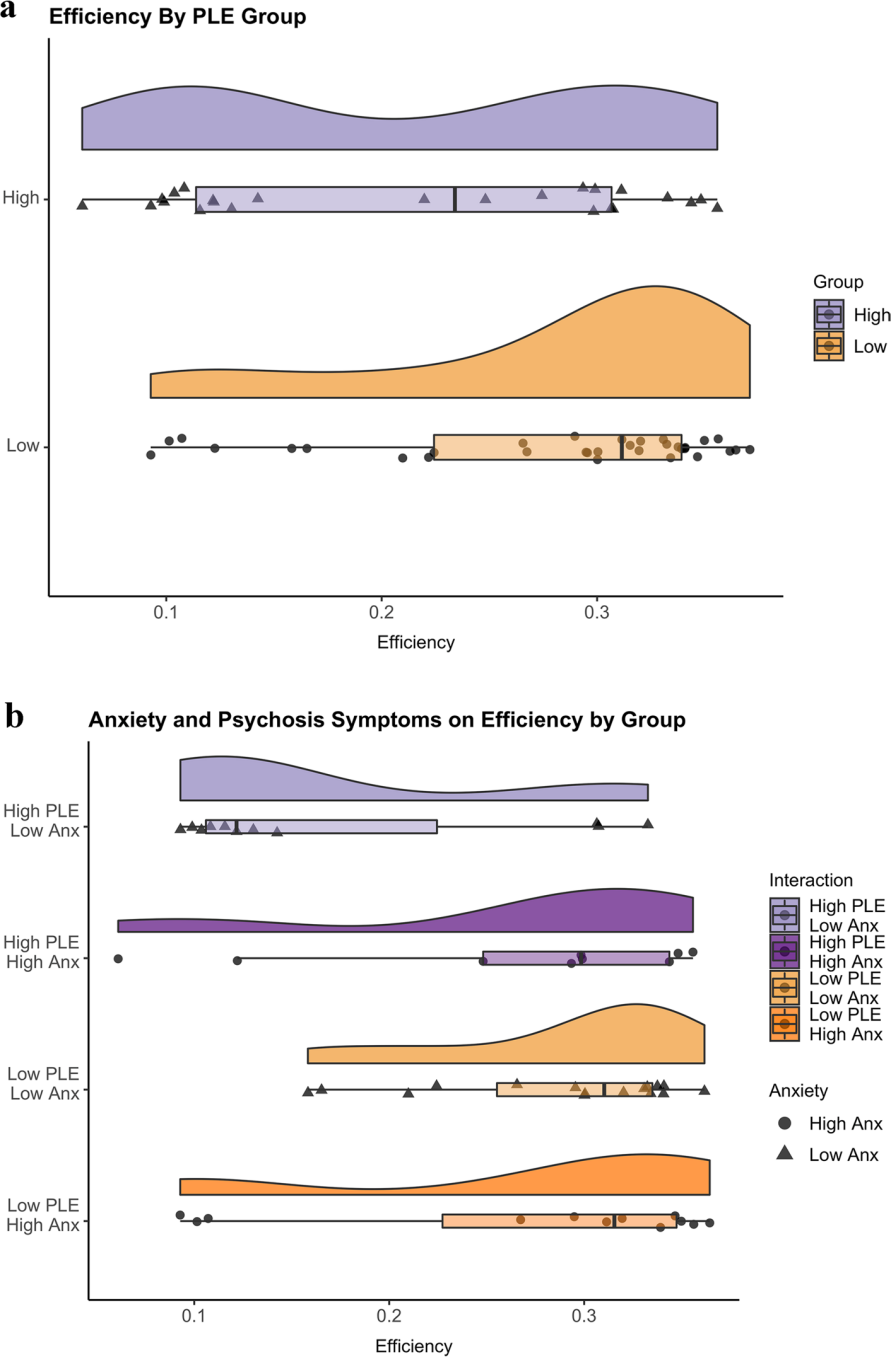
(**a**) Efficiency by PLE group. The high-PLE group made significantly less efficient responses compared to the low-PLE group. *PLE* psychotic-like experiences. (**b**) Efficiency relates to anxiety symptoms in the high-PLE group. Individuals with greater levels of anxiety in the high-PLE group made efficient responses comparable to those with low-PLE. Note that the PLE groups were divided into different high/low anxiety groups for illustrative purposes only and all analyses treated anxiety symptoms continuously. *Anx* anxiety symptoms, *PLE* psychotic-like experiences.

Efficiency was significantly different between the two groups, such that the high-PLE group was slower (*M* = 0.21, *SD* = 0.1) in selecting the disparate item than the low-PLE group (*M* = 0.28, *SD* = 0.08): *t*(55) = 2.59, *p* = 0.01, *d* = 0.69 (Fig. 3a).

#### Perseverative errors

**Figure 4:**
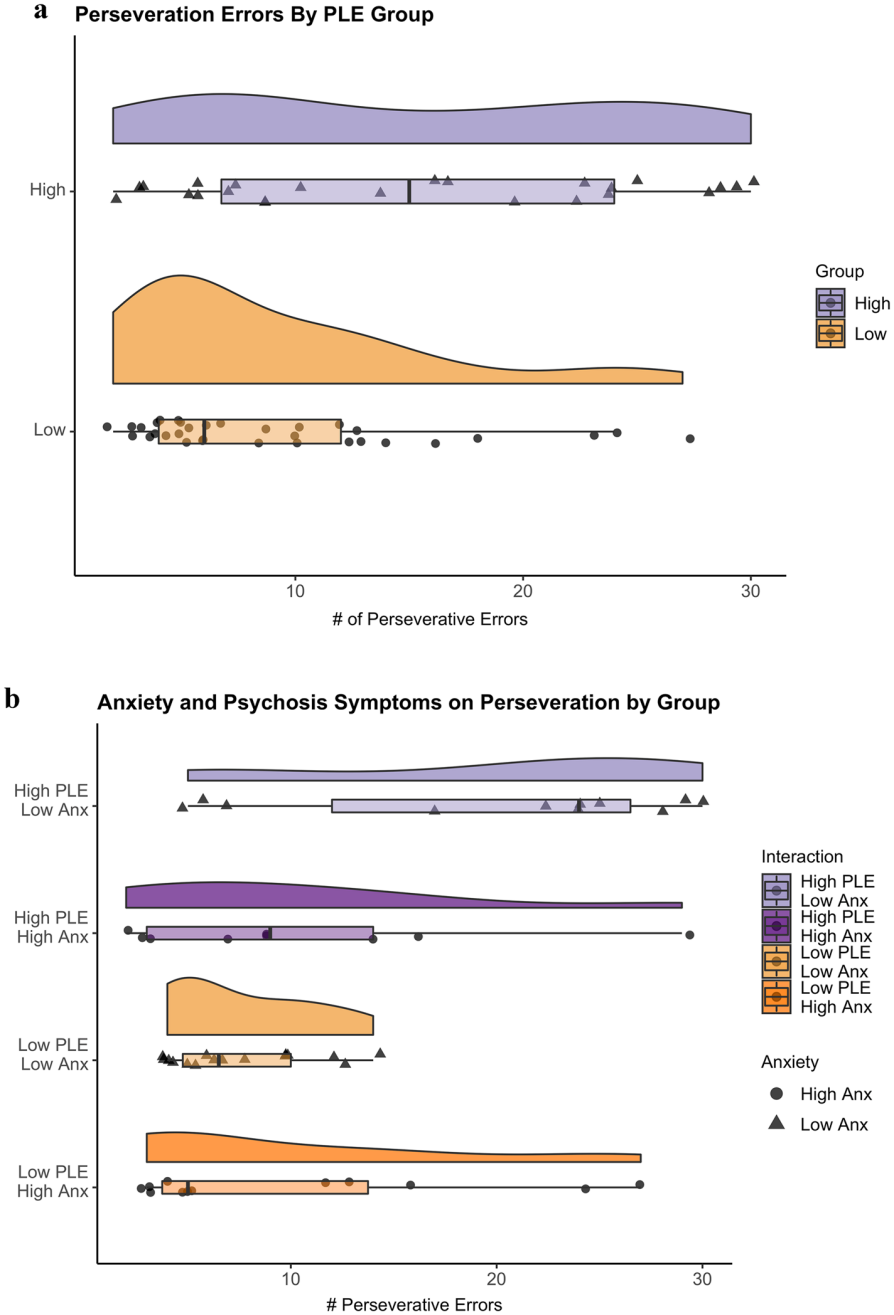
(**a**) Perseverative Errors by PLE group. The high-PLE group made significantly more perseverative errors compared to the low-PLE group. *PLE* psychotic-like experiences. (**b**) Perseverative errors relate to anxiety symptoms in the high-PLE group. Individuals with greater levels of anxiety in the high-PLE group made perseverative responses comparable to those with low-PLE. Note that the PLE groups were divided into different high/low anxiety groups for illustrative purposes only and all analyses treated anxiety symptoms continuously. *Anx* anxiety symptoms, *PLE* psychotic-like experiences.

The number of perseverative errors made throughout the task was significantly different between the two groups, *t*(55) = 2.93, *p* = 0.005, *d* = 0.79, such that individuals in the high-PLE group made more incorrect responses due to perseveration (*M* = 15.33, *SD* = 9.63) compared to the low-PLE group (*M* = 9.09, *SD* = 6.49) (Fig. 4a).

### PCET performance by non-clinical psychosis group and anxiety symptoms

#### Accuracy

There was a main effect of PLE group on accuracy: *F*(1,55) = 5.99, *p* = 0.01. There was no main effect of anxiety symptoms on accuracy. However, there was a significant interaction between severity of psychosis-like experiences and anxiety symptoms, *F*(3,44) = 4.35, *p* = 0.01, such that higher anxiety symptoms predicted better accuracy in the high-PLE group, and worse accuracy in the low-PLE group (Fig. 2b).

#### Efficiency

There was a main effect of PLE group on efficiency: *F*(1,55) = 6.69, *p* = 0.01. There was no main effect of anxiety symptoms on efficiency. However, the interaction between PLE and anxiety symptoms significantly predicted efficiency, *F*(3,44) = 6.76, *p* = 0.01, such that higher anxiety symptoms predicted better efficiency in the high-PLE group, whereas the opposite was true for the low-PLE group (Fig. 3b).

#### Perseverative errors

There was a significant main effect of PLE endorsement on perseverative errors, *F*(1,55) = 8.56, *p* = 0.004. Although there was no main effect of anxiety symptoms on perseverative errors, there was a significant interaction between psychosis-like experiences and anxiety symptoms, *F*(3,44) = 6.76, *p* = 0.001, where higher anxiety symptoms predicted less perseverative errors in the high-PLE group and more perseverative errors in the low-PLE group (Fig. 4b).

## Discussion

Individuals with elevated levels of psychotic-like experiences demonstrated increased symptoms of anxiety and deficits in executive functioning. These deficits were present across all facets of executive functioning measures: lower accuracy, lower efficiency, and more perseverative errors. Importantly, the pattern of association between PLEs and performance was divergent based on endorsed anxiety symptoms. In individuals with elevated levels of PLEs, anxiety symptoms seem to facilitate, rather than impair, cognitive functioning—those with heightened anxiety symptoms performed better than their peers with lower levels of anxiety. Conversely, in the low-PLE group, high levels of anxiety symptoms hampered optimal performance. Taken together, these findings provide preliminary evidence to suggest that the effects of co-occurring anxiety symptoms in individuals with non-clinical levels of psychosis may provide an important perspective for understanding the vulnerability on the lowest level of psychosis continuum.

The findings from the current study add to the growing literature on executive deficits in individuals with PLEs and suggests that executive functioning can be impacted by non-clinical levels of psychosis. Higher levels of PLEs were associated with less accurate responses and were slower in selecting the disparate item. Their difficulty with making the correct and efficient responses reflects deficits in rule learning. The high-PLE group was also associated with significantly more perseverative errors, despite the feedback which indicated that the principle has changed. These individuals were more likely to persist in adhering to the rule that was no longer in effect, suggesting impaired cognitive flexibility. Our findings are consistent with previous research which found that positive psychosis-like symptoms adversely impact some aspects of cognitive functioning such as inhibitory control^53,54^ and working memory^55^. Moreover, it has been observed that individuals with PLEs display abnormalities with cortical regions that are highly associated with executive functioning^56^. The present study extends this line of research, finding that PLEs are also negatively associated with rule learning and cognitive flexibility.

As expected, elevated symptoms of anxiety were observed in the high-PLE group compared to the low-NCP group. Unsurprisingly, individuals with early psychotic symptoms (e.g., first episode psychosis) report experiencing distressing levels of social phobia^57^ and panic symptoms^58^. Although the present study did not examine PLEs with a specific anxiety disorder, we observed that PLE elevations are predictive of greater emotional and somatic experiences of anxiety in our community sample. Moreover, the present study highlights that elevated levels of anxiety symptoms are still observed in healthy, non-help seeking individuals with PLEs.

In the exploratory analyses of the interactive effects of PLEs and anxiety symptoms, greater anxiety symptoms yielded better task performance in the high-PLE group. These individuals performed comparable to those who endorsed lowest levels of PLEs and anxiety symptoms. In contrast, individuals with high-PLEs and low anxiety symptoms performed significantly worse. Such results are consistent with observations from individuals with first episode psychosis^8^, who show benefits in executive functioning related to anxiety symptoms. Despite the debilitating effects of anxiety symptoms on cognitive functioning^21,22^, the present study suggests that in individuals with high levels of PLEs, anxiety symptoms may be beneficial to rule learning and cognitive flexibility.

We propose that such counterintuitive effects on anxiety symptoms in the context of PLEs may be supported by two existing theoretical models: the optimal arousal theory and the adaptation theory. The optimal arousal theory posits that a certain level of arousal is necessary for the most effective learning^29^, suggesting an inverted u-curve to explain the relationship between arousal and performance. Arousal in a community sample with non-clinical levels of psychosis has yet to be examined; nevertheless, physiological data suggests that individuals with sub-clinical psychosis exhibit a decreased parasympathetic activity while reporting an increased experience of stress during paranoia^59^. Such results provide evidence that anxiety symptoms may be providing some level of arousal necessary for optimal performance among high-PLE individuals. Future research should examine possible mechanisms of arousal in this effect, including whether high-PLE individuals experience arousal from anxiety that confers a benefit to executive function performance. That said, anxiety symptoms and arousal are different constructs, and the theory does not sufficiently explain what is being observed in the current study.

One possible alternative explanation is that increased anxiety symptoms are an adaptive response to a stressful mental state, and therefore may be reflective of intact cognitive functioning rather than its impairment^8,33^. Herniman et al.^33^ and Lindgren et al.^8^ hypothesize that individuals with a recent experience of PLEs but also with intact cognition have greater insight of their mental state, leading to increased feelings of worry and rumination. Relative to patients with a schizophrenia diagnosis, individuals on the lower end of the continuum may be more aware of their recent psychosis-like experience and cognizant of its development. As the adaptation theory suggests, while such heightened awareness may be associated with increased worry and affective symptoms^60^, early intervention during this period may lead to better treatment outcomes^61^. Thus, it will be important for future studies to examine whether the benefit of anxiety on executive function performance is mediated by patient insight.

The present findings underscore the importance of exploring the interaction between anxiety and psychotic symptoms and their relationship to emerging cognitive changes in non-clinical population. However, there are several limitations that should be considered for this study. Although the size of the PLE groups is comparable to those of other studies^62,63^, future studies would benefit from a larger sample that enables them to compare the anxiety levels within the PLE groups and interpret the mechanism underlying the observed effect of anxiety symptoms. Future studies should consider examining potential mediation effects, rather than interactions, to further examine the causal role of anxiety symptoms in PLEs and cognitive performance. The current study also relies on the Beck Anxiety Inventory as a measurement of self-reported anxiety. The BAI is a highly reliable questionnaire that has been proven useful for measuring anxiety in non-clinical populations^43,45^. However, the majority of the items measures somatic experiences of anxiety (e.g., numbness or tingling, feeling hot, wobbliness in legs) rather than cognitive and affective experiences^45^. Thus, cognitive features of anxiety such as rumination or mental rigidity may not be well-detected from the BAI. Moreover, despite the sensitivity of BAI to anxiety severity, questions of its validity have previously been raised^64^. As such, future work may consider the use of other measures of anxiety symptoms. Similarly, although CAPE is a valid and reliable measure of psychotic-like experiences in the general population, future studies should consider incorporating distress scores in addition to experience frequency. The present study focuses on specific domains of executive functioning. Future studies should examine broader cognitive abilities to understand whether anxiety symptoms and PLEs selectively impact certain domains. Finally, future work should consider incorporating physiological measures of arousal, in addition to self-report measures of anxiety symptoms, to further test the relationship between arousal and cognitive performance.

In conclusion, the present study highlights executive function deficits in psychotic-like experiences and explores the potential role of anxiety symptoms in the context of PLEs and their integrative effect on executive functioning. High levels of anxiety symptoms are common in PLEs, though their interaction may play a favorable role in cognitive flexibility and rule learning. This study emphasizes the importance of assessing non-clinical levels of psychosis in consideration with co-occurring symptoms.

## Supplementary Information

Supplementary Information.

## Data Availability

De-identified data is available upon request. All analyses were completed using RStudio version 1.4.1106. The R script used for analyses can be found on Github: https://www.github.com/jadynpark/NCP.
