# Supplementary material for: Anxiety symptoms, rule learning, and cognitive flexibility in non-clinical psychosis
Source: Sci Rep. 2022 Apr 5;12:5649. doi: 10.1038/s41598-022-09620-z (PMC8983653; doi:10.1038/s41598-022-09620-z)
Supplement: Supplementary file 1 — Supplementary Information. [file 41598_2022_9620_MOESM1_ESM.docx]

**Figure 1.** *PCET* *accuracy by PLE group and anxiety symptoms interaction.* There was no main effect of anxiety symptoms, but there was a significant interaction between severity of PLEs and anxiety symptoms.


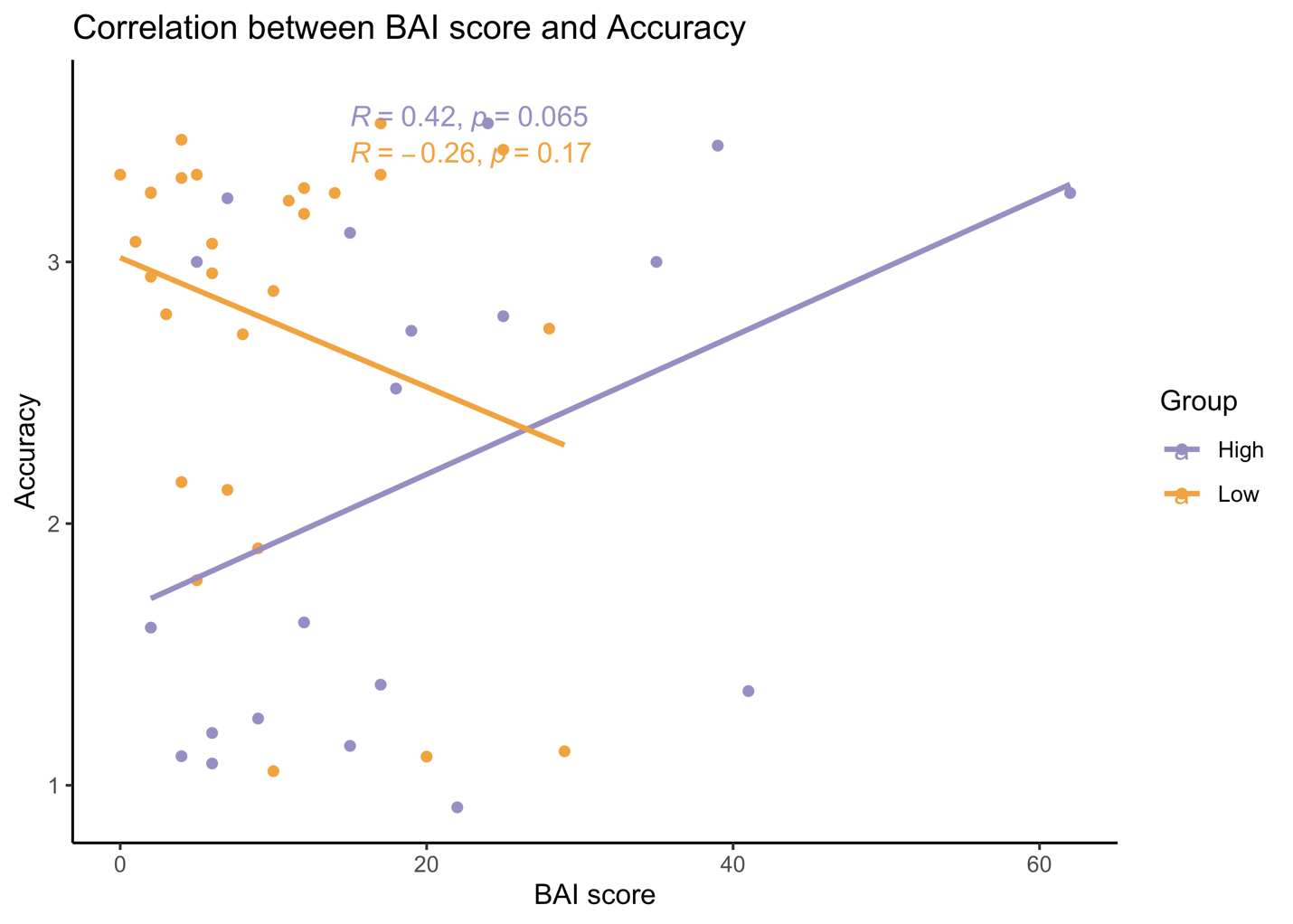


BAI: Beck Anxiety Inventory; High: high-PLE group; Low: low-PLE group

**Figure 2.** *PCET* *efficiency by PLE group and anxiety symptoms interaction.* There was no main effect of anxiety symptoms, but there was a significant interaction between severity of PLEs and anxiety symptoms.


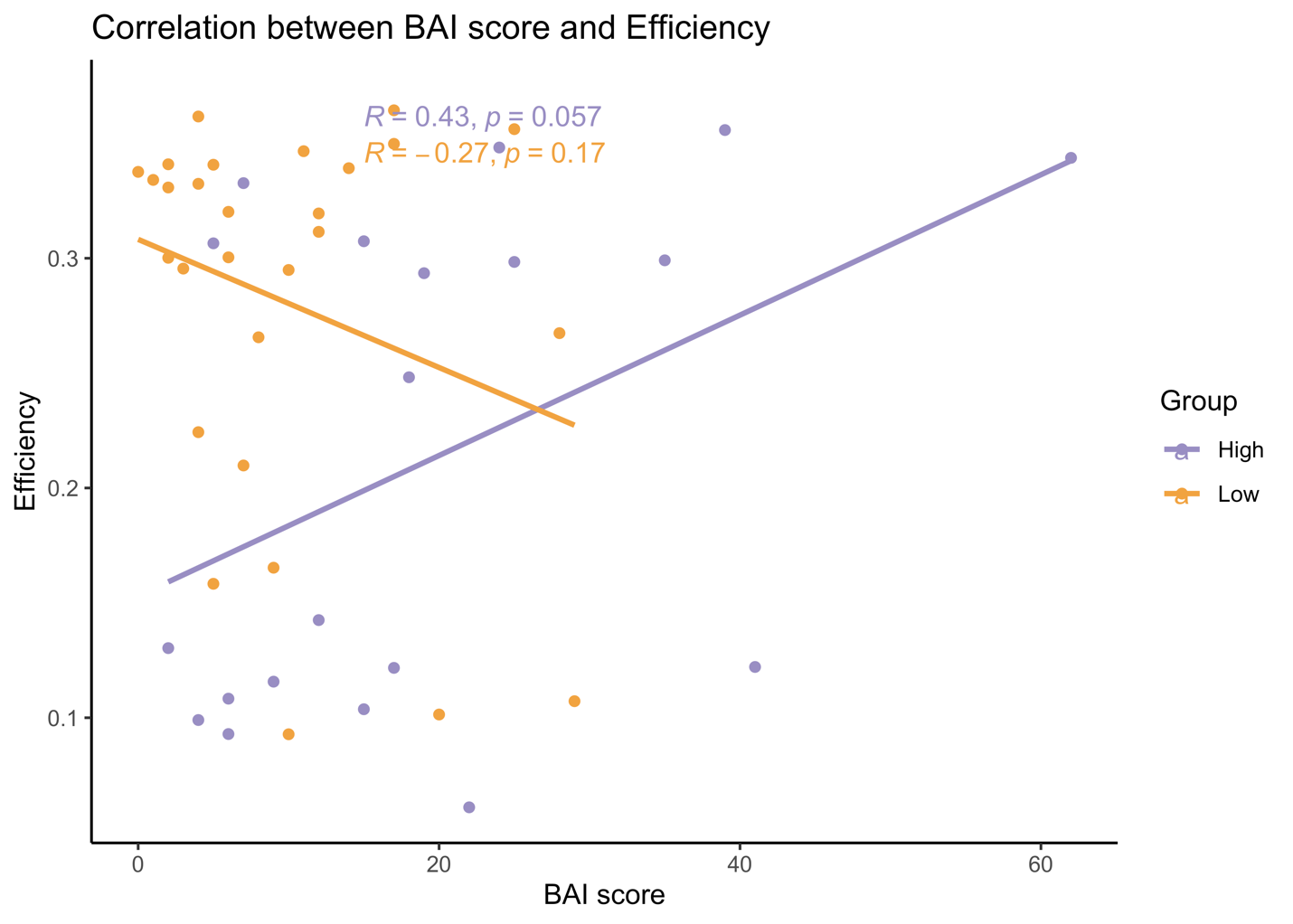


BAI: Beck Anxiety Inventory; High: high-PLE group; Low: low-PLE group

**Figure 3.** *PCET* *perseverative errors by PLE group and anxiety symptoms interaction.* There was no main effect of anxiety symptoms, but there was a significant interaction between severity of PLEs and anxiety symptoms.


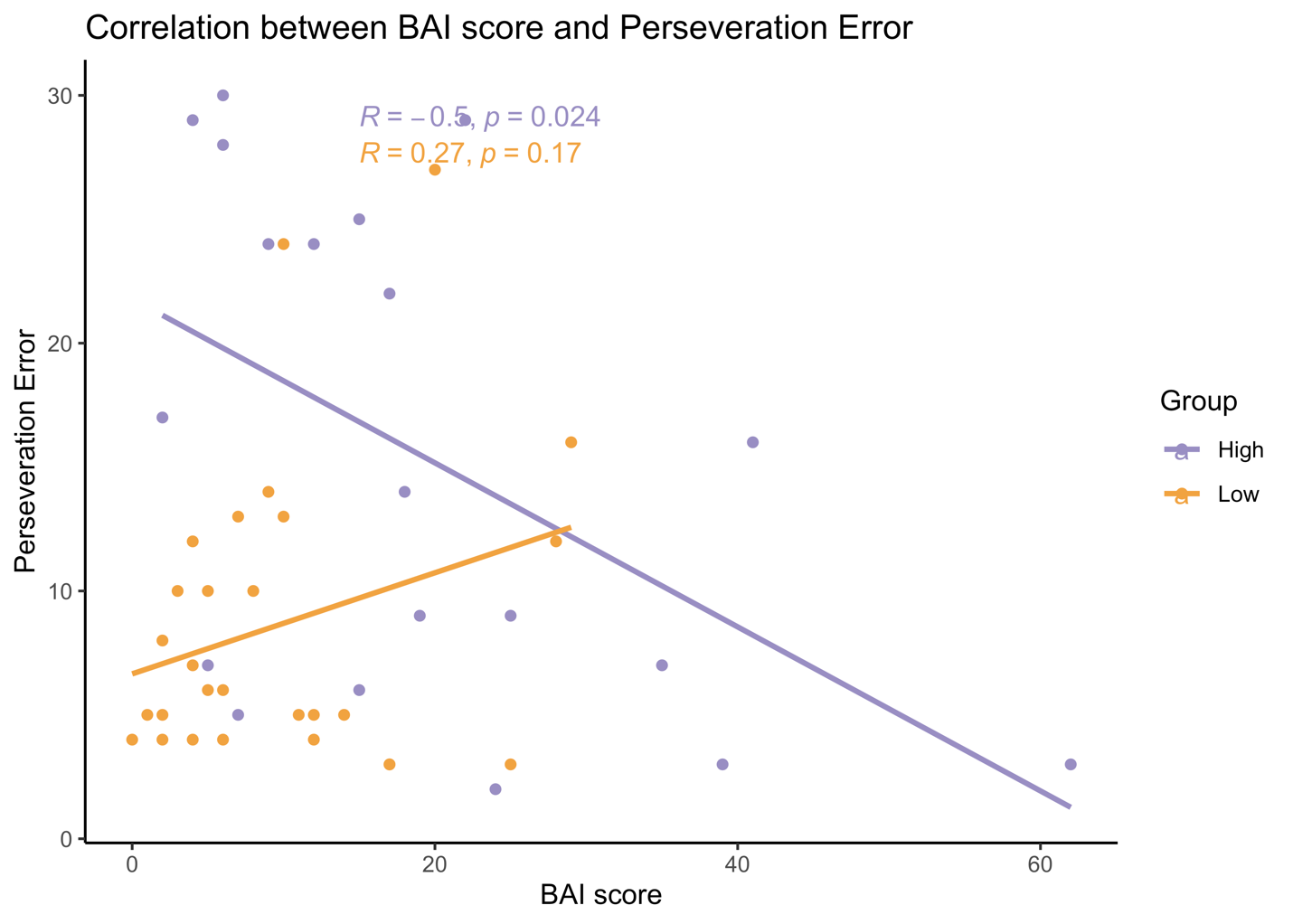


BAI: Beck Anxiety Inventory; High: high-PLE group; Low: low-PLE group
